# Mucoid or Puruloid Engorgement of the Antrum

**Published:** 1884-12

**Authors:** John S. Smith

**Affiliations:** Lancaster, Pa.


### SELECTIONS.

### Mucoid or Puruloid Engorgement of the Antrum.

BY JOHN S. SMITH, D.D.S., OF LANCASTER, PA.

Mrs. H., married, aged 24 years, was brought in consultation to me in August, 1883, by her dentist, Martin Musser, D. D. S., of this city. The patient had suffered for a period of nearly two years with neuralgia and periodontitis at times in her left upper jaw and face. The pain was at first felt in the pulp of the first bicuspid tooth; the pulp was subsequently devitalized, and the canals filled with a temporary filling. For certain reasons the operation was not completed with a permanent filling. The case came into Dr. Musser's care, who finally extracted the tooth, which gave no relief. The face continued swelling, the pain became unbearable, and it was concluded to consult other advice. I made a through examination of her mouth and teeth upon the affected side. The gums over and around the first and second molars were inflamed. These teeth were filled with large gold plugs, which occupied the crowns, and on percussion with an instrument showed some degree of pain. The wisdom tooth was missing, having not erupted. Suspecting diseased condition of the antrum, my attention was directed to the nares of the affected side, which was found free from moisture. There was noticed a discharge of a mucous nature escaping from the inner canthus of the eye; the conjunctiva was inflamed, with an intermittent flow of tears over the lower lid; the sight was somewhat defective at times. The patient showed symptoms of a marked anæmic condition; had lost in body weight; appetite poor; the pain was intermittent; when it was the most severe she located it under and back of the orbit, and in the body of the cheek bone; here the pain was characterized as a dull sensation, and at times producing a fullness as though the face would burst. Further examination revealed uniform fullness over the region of the antrum, and slight fluctuation upon pressure, and tender to the touch; the skin presented a blush-red appearence with a number of rose-red and purplish-colored spots scattered upon its surface. This condition was more noticeable when the pain was greatest, thus showing the deep-seated congestion of the parts involved.

*Diagnosis.*—The foregoing history of the case was sufficient to establish the fact of an engorgement of the antral cavity, brought about by the result of a reflected chronic periodontal inflammation. The tumor noticed upon the cheek was distinguished from others that might affect this particular locality: first, by the history of the case; second, by the dryness of the nares of the affected side; third, by the gradual and regular enlargement; fourth, by the non-association of the integuments of the cheek; and fifth, by a fluctuation which it yielded upon pressure. The long continued periodontal inflammation resulted in the engorgement of the antral cavity by a reflected chronic condition of the parts, which were filled with a profuse discharge and closure of the orifice or outlet of the sinus, thus causing the attenuated condition of its walls, which was the result of the protuberance noticed upon the cheek. One of the weakest points had finally given way; namely, a slight rupture into the orbit. From this fistula a muco-purulent fluid (inodorous) escaped. This established our diagnosis—mucoid, or purulant engorgement of the antrum.

*Treatment.*—"To treat successfully such a disease we have only to search out the source of offence, and where it is possible, remove it." The first superior molar was directed to be removed, which was done by Dr. Musser a few days later. The pulp was found to be dead. There was no discharge from the alveolus after its removal. Disappointed in this, an attempt was made to puncture the antrum through the palatine socket of the tooth. The pain being too great, further operation for the time was abandoned. A few days later the patient was again brought to my office, suffering greatly; the face was swollen, and she was feverish and the mouth was hot. These symptoms were controlled by proper remedies, and an external application of lead-water and laudanum was ordered to be applied, and the patient dismissed until the inflammatory condition had subsided. At the next sitting, a few days afterwards, the second molar was removed and the pulp also found dead; and there was some absorption of the alveolar process (buccal portion). Upon its removal, a small quantity of thin ropy fluid escaped with the blood. The outlet not being sufficiently large, the patient was etherized by Drs. D. R. Summy and Musser, and I passed a suitable-sized trocar through the alveolus of the palatine fang, opening into the antrum; this was followed by more abundant purulent discharge. The cavity was then thoroughly washed with tepid water, and the following stimulant wash prescribed:

| | | |
|---|---|---|
| ℞. | Glycerinæ | ℥ j. |
| | Tinct. opii camph | ℥ ij. |
| | Aquæ coloniæ | ℥ iv. |

M. S.—To be thrown into the cavity daily with a syringe.

This, in addition with phenoli sodique diluted and warm water, formed the local dressings, observing to keep the opening patulous with cotton-wool. The general systemic treatment consisted in taking good, nourishing food and free exercise in the open air, and the administration of copaiba, tincture of iron, and quinia. In six months the patient made a good recovery from the antrum trouble. The purulent secretion from the eye ceased, but at this writing the eye still remains weak and at times a profuse lachrymal flow over the lower lid, notwithstanding she has had the probe passed for stricture of the lachrymal duct. Hopes, however, are entertained, by the ophthalmic surgeon having this now under treatment, of a final cure.

---

The death of a child, aged two and a half years, recently occurred, in England, from the sting of a wasp on the arm. Severe inflammation of the limb set in, and in four days the fatal result took place.

